# Female mating tactics in lekking fallow deer (*Dama dama*): experience explains inter-individual variability more than costs

**DOI:** 10.1038/s41598-020-58681-5

**Published:** 2020-02-27

**Authors:** Simona Imperio, Sonia Lombardi, Annamaria De Marinis, Francesca Ronchi, Giacomo Santini, Stefano Focardi

**Affiliations:** 1 ISPRA (Italian National Institute for Environmental Protection and Research), Via Ca’ Fornacetta 9, I-40064 Ozzano dell’Emilia, BO Italy; 2 Department of Biology, University of Florence, Via Madonna del Piano, 6, I-50019 Sesto Fiorentino, FI Italy; 3 CNR-ISC (Institute for Complex Systems), Via Madonna del Piano, 10, I-50019 Sesto Fiorentino, FI Italy

**Keywords:** Behavioural ecology, Animal behaviour

## Abstract

Most studies on ungulate reproduction have focused on the covariates of male reproductive success, while there is much less information on female tactics of mate choice. The aim of this work is to fill this gap and to assess condition-dependent variations in female tactics in a lekking fallow deer (*Dama dama*) population. In particular, we investigated three indirect selection mechanisms: i) *aggregation:* when females join an already formed female group; ii) *copying*: when females copy the mate choice of other females and iii) *territory choice*: when females select a territory where many copulations had previously occurred. Our results show that female fallow deer, which are less experienced (younger) and/or incur higher travel costs (home range far from the lek), adopt indirect forms of mate selection more often than older females or females residing near the lek, respectively. Compared to adults, younger females remained longer in the lek (almost three times) and in male territories, returning to the lek after copulation. However, despite the time spent at the lek, younger females were not able to select the highest-rank males, and relied on *territory choice* more often than older females. Farther does visited the lek less frequently (farthest females only once) and arrived on average 5 days later than closer females (which performed up to 7 visits), but they were seen more often within female groups (*aggregation*). We did not find a different amount of *copying* in younger or in farther females. Our results contribute to advance our understanding of female behaviours in ungulate leks.

## Introduction

In sexual reproduction, mate choice is a behavioural tactic^1^ by which individuals of one sex gain higher fitness by preferring to mate with some specific individuals of the other sex. In most species, females are the choosy sex while males compete intra-sexually and this process results in sexual selection^2^.

Sexual selection attains extreme levels in lek breeding system where many males and females congregate in small areas, a situation that often leads to fierce male-male competition. In leks, males aggregate and defend small display territories usually located very close to one another and leks are typically characterised by a strong asymmetry in male reproductive success^3–6^. Females do not get any resources such as food or parental care from males except their genes, but some authors have argued that females might obtain direct benefits, such as a reduction of transmission of venereal diseases and ectoparasites, or the reduction of social interference^5,7–9^.

Male assemblages called “choruses” are observed in several acoustically advertising taxa such as anurans^10^ and orthopterans^11^. Lekking birds such as the ruff (*Philomachus pugnax*^12^), black grouse (*Lyrurus tetrix*^4^), and manakins (*Chiroxiphia lanceolata*^13^) differ in how competition and mate choice contribute to female fitness. A small number of ungulates are also known to form leks such as the fallow deer (*Dama dama*^14,15^), the topi (*Damaliscus lunatus*^16^), the sika deer (*Cervus nippon*^17^), the Uganda kob (*Kobus kob thomasi*^18^), the white-eared kob (*K. k. leucotis*^19^) and the Kafue lechwe (*K. leche kafuensis*^20^).

Females are often the choosy sex, with choosiness being defined as the effort a female is prepared to invest in mate assessment in terms of the numbers of potential males sampled, or time spent per male^21^. Janetos^1^ contrasted two main decision tactics: *best-of-n* and *fixed threshold* rules. The *best-of-n* decision rule is based on direct comparisons of males, which do not require the assessment of an absolute score for each individual encountered, while the *fixed threshold* rule requires that a female is able to evaluate male quality on an absolute scale.

Two types of costs constrain the efficiency of mate choice rules: direct costs (in terms of, e.g., energy consumed, predation risk) which can strongly reduce the net benefit of mate choice; opportunity costs appear when there is intra-sexual competition for the same mate, which can arise easily in case of sperm depletion. Among ungulates, the costs of female mate selection can be huge as observed in a pronghorn (*Antilocapra americana*) population where males defend harems^22^.

The opportunity to compare many males on the same stage leads females to visit leks^7^ because a reduction of direct costs may enhance female choosiness. Thus it is not surprising that female choice plays an important role in the evolution of leks. A female makes a direct assessment when, after having visited many males, chooses one of them exclusively on the basis of his phenotype^23,1^. However, mating in a lek generates an asymmetry of costs among females, since females living close to the lek area benefit by lek breeding more than females living far from the lek. This is because (i) energetic expenditure to get to the lek and (ii) predation (or accident) risk during the displacement are larger, while (iii) the time left for other activities, such as foraging^24^ is lower for females living far from the lek. Thus, we expect that females which incur higher costs, would adopt secondary mate choice tactics that allow them to reduce the time necessary for the evaluation of partner.

Secondary tactics of mate choice occur when a female chooses a partner using cues other than male phenotypic traits. This approach, described in several lekking species^25–27^, may allow them an efficient choice at reduced costs. Moreover, these tactics can be convenient for unexperienced females to increase the precision of mate choice^25^. Importantly, secondary tactics have the potential to increase the asymmetry observed in male reproductive success^28,29^.

In the following we consider three secondary tactics.

### Aggregation

Clutton-Brock *et al*.^30^ found that the number of females increases in territories where a harem is already present: females could aggregate (regardless their position in the arena) in order to have higher probabilities to join a successful male.

#### Copying

A female mates with a male that was previously observed to mate with other female(s)^31^.

#### Territory choice

A female chooses a male that is defending a territory where other copulations have taken place earlier^32^.

The main difference between *copying* and *territory choice* is that the latter uses also indirect cues, such as pheromones or territorial marks, while copying relies only on the direct observation of copulations. *Aggregation* differs from *copying* because it may allow females to realise quickly the position of successful males in the lek, even without observing copulations.

The fallow deer is an ideal species to study lek mating given that this cervid forms large reproductive aggregations, individual behaviour can be easily recorded, and males are easily identifiable by antler shape even in the absence of tags. Most previous field studies concerning leks of fallow deer focused on the covariates of male reproductive success (see^33^ and references therein), while there is much less information on female tactics of mate choice^9,14^; for non-lekking populations and controlled experiments see also^34–38^. As sexual selection is a co-evolutionary process between the two sexes, disregarding variation in females overlooks a key aspect of this process^39^.

**Figure 1:**
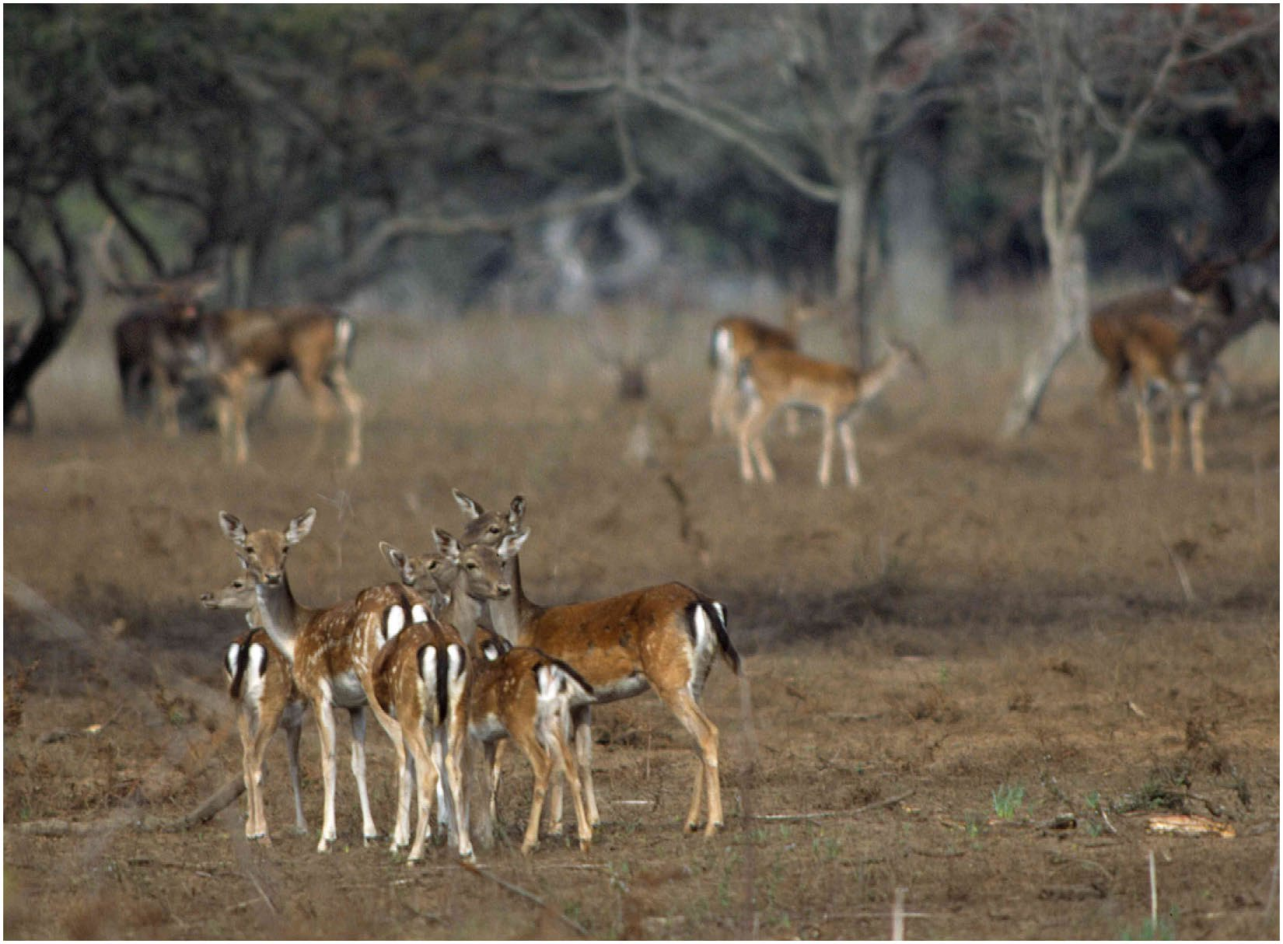
A group of female fallow deer attending the lek of Castelporziano (Italy). Photo by A. De Marinis.

Our paper aims to fill this gap of knowledge using the availability of information on the behaviour of a number of individually-marked females in the lek of Castelporziano (Italy) (Fig. 1). We aimed at verifying whether reproductive costs and/or experience affect the adoption of a particular tactic of mate choice in female fallow deer. More specifically, we assessed whether females were more likely to make use of direct assessment or secondary mating tactics in relation to both the location of their home range with respect to the lek (displacement costs) and their age (experience). Three main hypotheses have been developed for this analysis:

H1 *Direct mate assessment*. Females with no or reduced travel costs, or more experienced ones, should adopt, more often, a direct assessment tactic compared to younger females or those living far away. More specifically, we predict that females using a direct choice tactic are present in the lek before they copulate and perform a large number of visits in order to observe displaying males and evaluate their quality. We therefore expect that (a) the number of female visits to the lek is larger and the date of arrival is earlier for females whose home range is close to the lek. Consequently, we also expect closer females (b) to visit more territories and to spend more time in the vicinity of males. We also predict that (c) adult, and hence more experienced females will make less visits to the lek than younger females, who should stay in the lek for a longer time, and consequently (d) visit more territories to observe mating behaviour. This makes sense if they visit the lek when the mating activity is well developed and so we do not expect (e) that they arrive at the lek before adult females. We also expect that (f) near and experienced females will copulate earlier than far and younger females. Thus (g) the asymmetry in male mating success should increase over the course of the mating season. Females do not need to spend any time at the lek after mating, except if this is useful for gaining experience, thus we expect that (h) younger females will return to the lek after copulation more often than adults.

H2 *Secondary mating tactics* *allow reduced mating costs for females*^26,27^. Such tactics could be also used by less experienced (younger) females that are not able to make good choices on their own. We thus expect that females with higher travel costs, or younger females, are more likely to be (a) observed within female clusters (*aggregation*), and/or (b) to mate with a male that copulated immediately before her own copulation (*copying*) or (c) mating in a territory where more copulations had occurred earlier (*territory choice*).

H3 *Secondary tactics could increase the precision in mate assessment*^25,40^. If this holds true, and H2 predictions are verified, farther and younger females should make a better choice (mating with a highly successful male) than the ones that make a direct assessment. According to Losey *et al*.^41^, we are able to reliably use the total mating success of a male as a proxy for its quality.

## Methods

### Study area

The study was performed at Castelporziano in the years 2000–2003. The study area is a fenced reserve of 60 km^2^ located near Rome (Italy) (41° 44′N, 12° 24′E) (Fig. S1). The climate is Mediterranean, with dry summers and rainfall occurring primarily in October-November. The plant communities mainly consist of holly oak (*Quercus ilex*, 27%) and deciduous oak forests (*Q. cerris* and *Q. frainetto*, 34%), often associated with undergrowth of Oriental hornbeam (*Carpinus orientalis*, 80–90%). A number of domestic pine (*Pinus pinea*) stands were also present. A very detailed description of the vegetation of the study area can be found in^42^. Information on ungulate populations is found in^43^.

The lek was located in an open area of about 0.7 km^2^, characterized by the presence of an arid pasture (Fig. S1). This grassland is characterized by the presence of a few large oaks and a sparse undergrowth with common hawthorn (*Crataegus monogyna*), ferns (*Pteridium aquilinum*) and asphodel (*Asphodelum microcarpus*). The area lacked large predators, and culling was forbidden during the rut. Rangers reported that fallow deer have formed a lek in this area at least since the early 1980s.

### Data collection

We performed animal handling according to the present regulations related to animal welfare and with constant veterinary assistance. Authorization to capture was made according to Article 4 and 7 of the Italian Act on Wildlife 157/92, within the Agreement n. 411 of 12/5/1995 between the Italian National Institute for Environmental Protection and Research and the Segretariato della Presidenza della Repubblica. Upon capture, all animals were fitted with a soft mask (to reduce distress), which allowed deer to breathe normally. Deer were positioned on the right side to avoid ruminal meteorism, which might reduce respiratory efficiency. Neonates were handled wearing gloves. No sedation was used and handling time was kept as short as possible (within 10–15 minutes).

Several methods of capture were used. We carried out 24 net drives during winters (January-February) 2000–2002. Moreover, during the annual trapping season of wild boar, in August-September 1996–2002, some fallow deer (mainly 3–4-month old individuals) were captured accidentally. A small sample of neonates was captured in June 2000–2003 and marked with ear tags. A total of 123 females were ear-tagged (75 of which were observed, at least once, at lek as yearlings or adults in 2000–2003) and 9 adult females were also fitted with VHF radio-collars (Wildlife Materials HPLM 21100, Murphysboro, IL, USA). The overall weight of a radio-collar was 400 g, corresponding to about 0.007–0.012% of the weight of adult females. We observed no changes in the behaviour of animals with ear-tags or radio-collars during observations at the lek, during yearly deer counts carried out in spring, and other occasional encounters.

Radio-tracking was performed using ATS Rx1000s (Isanti, MN, USA) and Lotek Suretrack 2000 (Newmarket, Ontario, Canada) receivers with a three-element Yagi antenna. Outside the rut, we collected a fix every 24–48 hours (at least 12 fixes per month, homogenously distributed over day and night) while during the rut we increased the sampling rate. Depending on the available personnel, a different sampling design was adopted during the different years of study. In 2000, we took about 1 fix every four hours, with a higher frequency when the animal was moving. In 2001 we took a fix every 12–13 hours, if animals remained in the same area, but we used a four-hour schedule if the animal moved away from the previous location. Finally, in 2002 and 2003 fixes were taken every 24–25 hours but, using a non-directional antenna, we also recorded every three hours the arrival/departure of animals to/from the lek and in this case one or more supplementary fixes were taken.

Out of 75 females observed in the lek, 42 (captured as fawns or yearlings) were of known age. Note that all radio-tracked females were of unknown age. We classified does (females > 1 y.o.) of known age as:

1. Yearlings (15–16 month old) and prime-aged adults (from 27–28 to 87–88 months), indicated for short as 1.5, 2.5, …, 7.5 years (we refer to this classification as *Age7*).
  When we also included in the analysis the does of unknown age, we used three different classifications, depending on the analysis (see below):
2. Yearlings and adults (referred to as *Age2A*).
3. Yearlings + young adults (2.5 years old), other adults (*Age2B*).
4. Yearlings, young adults (2.5 years old), other adults (*Age3*).

For each animal we computed the distance between the centre of its home range (outside the rut) and the lek and we considered this value as a proxy for costs borne by females to mate in the lek.

The centre of the home range in spring-summer (immediately before the onset of the rut of the same year) was computed using the best available information. When possible (for 25 out of 27 radio-marked females/year, and for 16 out of 127 ear-tagged females/year), we computed the home range centre of the same year as observations. For radio-marked animals we computed the barycentre of March-August fixes. For ear-tagged females we used, as proxy of home range centre, the barycentre of available observations. Observations were collected mainly during spring counts (April^44^), and summer counts of wild boar (July-August^45^). When the observations of the same year were not available, we considered the last observation available or the capture location. We assumed a strict philopatry of female fallow deer because the radio-tracking data have shown only a single significant shift of the spring-summer home range between two years. We also observed that yearling females almost never left the maternal home range (Imperio *et al*. in prep.). Because of the limited precision of the computed distances we approximated these values using 1 km classes of distance binned from 0.5 to 8.5 km (*Dist*).

During the rut a variable number (1 to 4) of operators observed the behaviour of fallow deer at the lek. The observers were equipped with binoculars (*Zeiss 10* × *40,* *Vixen 20 × 80* and *Swarovski* *30 × 75*) and telescopes (*Swarovski 60x*) to be able to read the tags. To check most of the lek area operators used a blind and/or a high seat depending on the position of display territories. Observations were performed *ad libitum* from dawn to dusk.

The survey performed in 2000 has to be considered a pilot study since observations lasted from the 1^st^ to 16^th^ of October and only the presence of tagged animals and the number of copulations were recorded. Therefore, data from observations carried out in 2000 were not included in the analyses.

In 2001–2003 a standardized sampling protocol was adopted. Behavioural observations were carried out beginning the 20^th^ of September until the end of October. The arrival of the first female groups in the lek was considered as the starting date of the rut. The end date was identified as the day after the last observed copulation. The great majority (at least 90% each year) of bucks, i.e. males ≥ 4 y.o., holding a lek territory were individually identified by antler morphology^33^. Each hour we recorded the position and sex/age class of every animal inside the lek (hourly counts). The location and the association of tagged females with territory holders or other females were recorded in 2001 as often as possible, depending opportunistically on available observers and tasks to be performed, while in 2002–2003 we tried to record females’ movement continuously. We noted territory and buck identity of all copulations of both tagged and non-tagged females.

Every year we recorded the daily number of copulations and we distinguished between courtships ending with ejaculation (*Ejac*) and interrupted courtships (*IntC*). Since the yearly distributions of copulations were characterised by multiple modes, we used the median date of copulation as a measure of copulatory peak (*Peak*), and we divided the rut into *pre-Peak* and *post-Peak* periods. We also computed the overall rank of each buck and territory from the distribution of copulations recorded for each buck or territory. Specifically, ranks were from 1 to 5 (bins: >22, 10–22, 4–9, 1–3 and 0) for bucks and from 1 to 4 for territories (bins: >10, 6–10, 1–5 and 0). The variables used in the analyses are listed in Table 1, with a brief description for each of them. The scale of the variables is always at level of (female) individual/year except for *Cop_preP* and *Cop_postP*, which are computed at the level of (buck) population/year and *Clust* that is computed at the level of single observation occasion (of females).

**Table 1:** Descriptions of dependent variables.

Variables	Descriptions	Sampling years	Selection criterion
*Cop_preP*	Number of copulations before *Peak*	2001–2003	≥5 obs
*Cop_postP*	Number of copulations after *Peak*	2001–2003	≥5 obs
*Visits*	Number of visits of female at lek	2000–2003	
*Date_arr*	Date of first arrival at lek (days from *Peak*)	2000–2003	
*Date_cop*	Date of copulation (days from *Peak*)	2001–2003	*Ejac*
*Visits_after*	Number of visits at lek for females after copulation	2001–2003	*Ejac*
*N_Terr*	Number of territories visited by a female	2002–2003	≥3 min
*Time_terr*	Total time (min) spent in display territories	2002–2003	≥3 min
*Time_buck*	Total time (min) spent with bucks	2002–2003	≥1 min
*P_Cluster*	Probability to be observed together with other females	2001–2003	≥2 obs
*Clust*	Binary variable: 0 = alone, 1 = in group of females	2001–2003	1^st^ obs
*Male_cop_day*	Number of copulations of the buck, with which the female mated, from the dawn of the same day until the mating with the female	2001–2003	*Ejac* + *IntC*
*Male_cop_before*	Number of copulations of the buck, with which the female mated, from the beginning of rut until the mating with the female	2001–2003	*Ejac* + *IntC*
*Terr_cop_day*	Number of copulations observed in the territory, where the female mated, from dawn of the same day until the mating with the female	2001–2003	*Ejac* + *IntC*
*Terr_cop_before*	Number of copulations observed in the territory, where the female mated, from the beginning of rut until the mating with the female	2001–2003	*Ejac* + *IntC*
*Male_cop_tot*	Total copulatory success of the buck with which the female mated	2001–2003	*Ejac* + *IntC*

We report the years used for computing the different variables and the criterion used for inclusion in the analysis. Obs = observations; *Ejac* = number of copulations ending with ejaculation; *Ejac* + *IntC* = total number of courtships; *Peak* = peak date.

The date of arrival in the lek, *Date_arr* and the number of visits to the lek, *Visits*, were computed using different field data for radio-tracked and ear-tagged females, consequently are not directly comparable. For ear-tagged females observed at lek, observations in two consecutive days were ascribed to the same visit only if the time lag (in hours) between observations was lower than the average visit duration computed for radio-tracked females.

The rationale behind using different metrics for the copulatory success of a buck territory (Table 1) is as follows. Since the average duration of a visit to the lek by adult females is about two days, if the female uses *copying* or *territory choice*, relying on visual cues, we expect that the number of copulations happened on the same day (*Male_cop_day*, *Terr_cop_day*) are those of interest. If the female uses indirect cues inside the territory, such as marking, then the whole previous period would be relevant (*Terr_cop_before*). Moreover, some females could stay longer than average in the lek, therefore both *Terr_cop_before* and *Male_cop_before* could have some importance. Instead, in the case that a female mates with a buck on the basis of its phenotype we expect that male overall success (*Male_cop_tot*) should be relevant. For these analyses we used both *Ejac* and *IntC* because, irrespective of the outcome, any courtship indicates a mate selection by the female. Note that for variables involving copulations (*_cop and *Visits_after*) in case of multiple copulations by the same female we used only the first one observed.

### Statistical analysis

We compared the variance of *Cop_preP* and *Cop_postP* (number of copulations before and after *Peak*) to investigate whether the unanimity of female choice increased during the rutting season. We selected only bucks observed ≥5 times on lek during the hourly counts to avoid artificially increasing the number of zeros. Variances were compared using the Fisher’s test (PROC TTEST of SAS 9.4)^46^.

The remaining variables (Table 1) where used singularly as response variables of a series of models testing the effect of both distance from lek and age of females. Since some females were observed in several years we used generalized linear mixed models (GLMM)^47,48^ using PROC GLIMMIX of SAS 9.4^46^ including female identity as a random factor. Because many dependent variables were not normally-distributed, the appropriate distribution was selected^48^ (see Results).

For *Clust*, i.e. the occurrence of finding a newly arrived female in the lek grouped with other females, we used a binary distribution; for this variable only we also tested the effect of the ranks of bucks and territory to disentangle the effect of aggregation among females *per se* and the attractiveness of successful bucks and territories.

## Results

### Lek dynamics

Copulatory success of bucks was highly asymmetric but the degree of monopolisation varied from year to year. Only a fraction of the bucks (36.8–44.8%) were observed to mate, and the ten most successful males were responsible for 49.5–62.6% of all the copulations at the lek every year (Table 2). The median date of copulations (*Peak*) is also reported in Table 2 for each year, together with the variance of the male reproductive success for the pre- and the post-*Peak* periods (see also Fig. S2). It is evident that the variance of male copulatory success was larger in the post-*Peak* period.

**Table 2:** Total number of identified bucks (Tot), total number of reproductive bucks (Rep), number of copulations ending with ejaculation (*Ejac*), total number of courtships (*Ejac + IntC*), *Peak* date (October), standard deviation (SD) of male copulatory success during the pre-*Peak* and post-*Peak* phases, and results of equality of variances test (Fisher’s test) (prediction H1g).

Year	Tot	Rep	*Ejac*	*Ejac* + *IntC*	*Peak*	SD pre-*Peak*	SD post-*Peak*	F	*p*	High
2000	—	—	116	148	11	—	—	—	—	—
2001	133	54	244	319	11	2.88	3.84	1.78	0.007	49.5
2002	114	42	197	243	13	3.54	4.94	1.94	0.006	62.6
2003	172	77	445	638	15	2.28	4.84	4.51	<0.001	50.2

The test was not performed in 2000 since bucks were not individually identified. Last column reports the % of copulations performed by the 10 most successful bucks (High).

**Figure 2:**
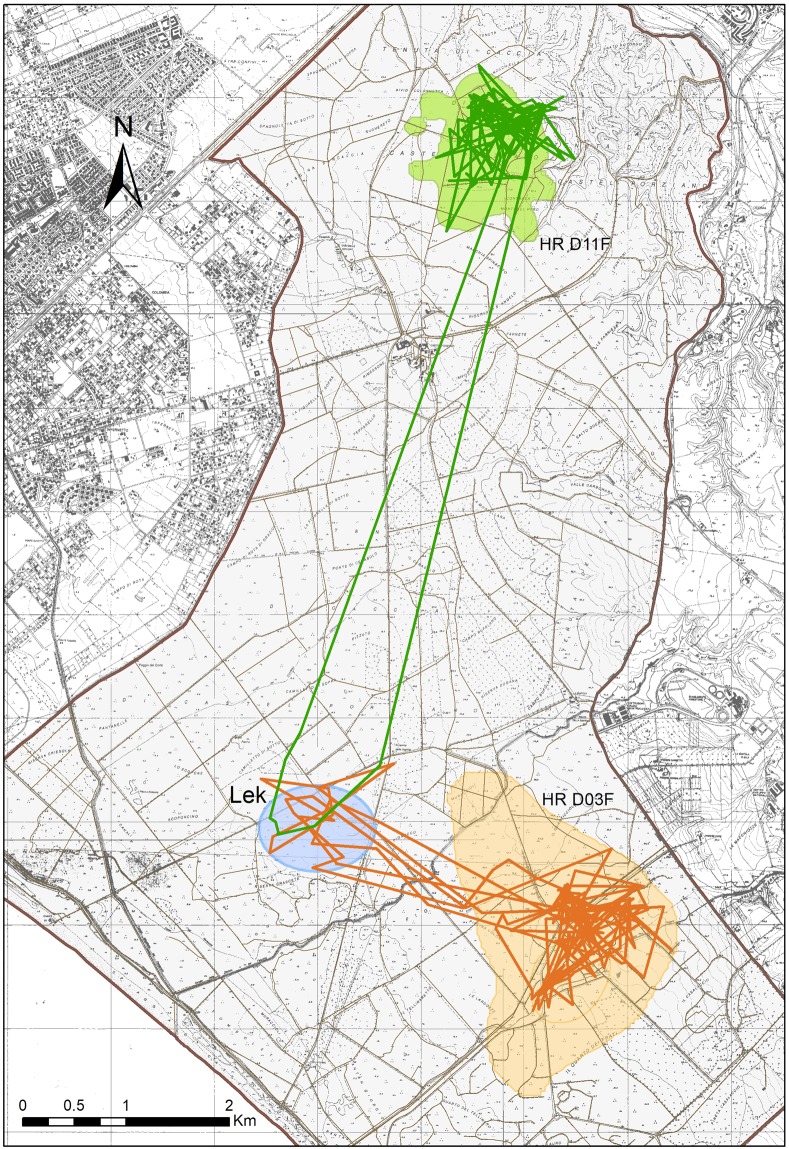
Paths of two radio-collared females (green: D11F, *Dist* = 7.5 km; orange: D03F, *Dist* = 2.5 km), performing one and three visits to the lek, respectively, during the breeding season in 2000 in the Castelporziano Preserve, Italy. Light orange and green areas represent the spring-summer home ranges of the two females, while the blue ellipse encloses the lek area (see Fig. S1). Map produced with ArcGIS 10.2.2. Background image is the Carta Tecnica Regionale 1:10000 (Lazio Region, 1990–1991), downloaded from http://dati.lazio.it/catalog/it/dataset/carta-tecnica-regionale-1991 under a Creative Commons Attribution License (https://creativecommons.org/licenses/by/4.0/deed.it).

Unlike bucks, which are present in large numbers in the lek well before the beginning of matings, females are abundant only around the *Peak* (Fig. S3). Almost all radio-tracked females visited the lek every year: only the farthest female (D11F, distance class: 7.5 km) visited the lek (once) in 2001 and 2003, but not in 2002 (Fig. 2).

During 2001–2003 we observed the mating of 29 tagged females (plus the interrupted courtships of other 6), of which three copulations were with an unknown buck, and two in an unrecorded territory. The large majority of females (90%) mated only once, but two of them were seen to mate twice (one yearling in the same day in 2002, and one 2-year old female after 4 days in 2003), and one 2-year old female mated three times in 2003 (the second time after 20 days, third time in the same day), all of them with different bucks.

#### H1. Direct assessment

The radio-collared females whose home range was closer to the lek visited the lek more frequently (up to 7 visits; Model M1, Table 3; Fig. 2) and arrived earlier (on average the females with distance classes 0.5–1.5 km arrived 5 days before females of classes 6.5–7.5 km; M2, Table 3). Note that the average length of a single visit (20.46 ± 2.76 hours) was not dependent on the number of visits (Kruskal-Wallis test: *H* = 5.42, *p* = 0.49). Thus overall lek attendance (up to 208 hours) is larger in close females (−0.76 ± 0.21 log[*Dist*], *p* = 0.002). In case of multiple visits, the average interval between successive visits was 4.24 ± 0.42 days.

**Table 3:** Results of Generalized Linear Mixed Models performed to characterise behaviour at the lek and mate choice of female fallow deer in relation to age and distance between home range and lek.

Model	Description	Sample	Hp	Type	N	Distance	*p*(*Dist*)	Age	*p*(*Age*)
***Direct assessment***									
M1	*Visits* ~ *log(Dist)*	Radiocollared does	H1a,c	Poisson	27	−0.65 ± 0.15	< 0.001	—	—
M2	*Date_arr* ~ *log(Dist)*		H1a,d	Normal	26	3.78 ± 1.10	0.003	—	—
M3	*Visits* ~ *log(Dist)* + *Age7*	All marked does	H1a,c	Poisson	149	−0.29 ± 0.10	0.006	−0.17 ± 0.06	0.008
M4	*Date_arr* ~ *log(Dist)* + *Age7*		H1a,d	Normal	149	2.31 ± 1.00	0.03	0.25 ± 0.57	NS
M5	*N_Terr* ~ *log(Dist)* + *Age7*		H1b	Poisson	55	−0.15 ± 0.13	NS	−0.36 ± 0.07	< 0.001
M6	*Time_Terr* ~ *log(Dist)* + *Age7*			Normal	55	−34.2 ± 36.3	NS	−44.3 ± 19.7	0.04
M7	*Time_buck* ~ *log(Dist)* + *Age7*			Normal	59	−12.55 ± 15.82	NS	−20.3 ± 8.63	0.03
M8	*Date_cop* ~ *log(Dist)* + *Age3*	All marked does, copulation observed	H1f	Normal	29	−0.73 ± 1.21	NS	−4.63 ± 0.86	0.03
M9	*Visits_after* ~ *log(Dist)* + *Age2B*		H1h	Poisson	29	0.071 ± 0.30	NS	−0.97 ± 0.30	0.045
***Aggregation***									
M10	*P_cluster* ~ *log(Dist)* + *Age2A*	All marked does	H2a	Logit	111	0.23 ± 0.13	NS	−0.01 ± 0.17	NS
M11	*Clust* ~ *Dist*^*(1)*^ + *Age2A*			Binary	111	0.24 ± 0.11	0.04	0.76 ± 0.53	NS
***Copying***									
M12	*Male_cop_day* ~ *log(Dist)* + *Age3*	All marked does, copulation observed	H2b	Poisson	32	−0.46 ± 0.36	NS	0.33 ± 0.39	NS
M13	*Male_cop_before* ~ *log(Dist)* + *Age3*			Poisson	32	−0.34 ± 0.41	NS	0.47 ± 0.31	NS
***Territory choice***									
M14	*Terr_cop_day* ~ *log(Dist)* + *Age3*	All marked does, copulation observed	H2c	Poisson	33	−0.14 ± 0.48	NS	−0.61 ± 0.44	NS
M15	*Terr_cop_before* ~ *log(Dist)* + *Age3*			Poisson	33	0.13 ± 0.35	NS	−1.32 ± 0.21	< 0.001
***Mate quality***									
M16	*Male_cop_tot* ~ *log(Dist)* + *Age3*	All marked does, copulation observed	H3	Poisson	32	−0.19 ± 0.26	NS	0.84 ± 0.20	0.005

Codes for hypotheses/predictions follow the list given in the Introduction.

(1) We used *Dist* and not *log(Dist)* since this model was considered more parsimonious (ΔAICc = 1.82).

Considering the larger sample of all the tagged females, we confirm the previous results relative to radio-tracked females in relation to distance (M3-M4, Table 3). From models M3-M4 we can also deduce that younger females were more prone to visit (yearling females made up to 16 visits, on average 3.76 ± 0.69 vs. 1.55 ± 0.18 visits of female aged 4.5–7.5 years; M3, Table 3), staying longer (yearling females were observed 17.04 ± 3.27 hours in the lek vs. 5.94 ± 1.03 hours of female aged 4.5–7.5 years) but they did not arrive in the lek before adult females (M4, Table 3).

If we consider the female behaviour at the lek in more detail, we found that younger females visited a higher number of territories (M5, Table 3), spent more time in display territories (M6, Table 3) and in proximity to a territorial buck (M7, Table 3), than older females. Contrary to our expectations, none of these variables were related to distance (M5-M7, Table 3).

Contrary to our predictions, there was no relation between copulation date and distance, while we found that younger females mated later than adult females (M8, Table 3). If we pool yearlings with 2-year old females, the number of visits in the lek after mating was higher for the younger class, while it did not depend on the distance (M9, Table 3). A larger fraction of yearlings (83.3%) than either the 2-year old (50%) or older females (21%) returned to the lek after mating (χ^2^ = 7.80, *p* = 0.020). This result remains valid even pooling yearlings and sub-adults to be compared to adults (χ^2^ = 4.75, *p* = 0.029).

#### H2. Secondary mating tactics

Interestingly, females living far away were more likely to stay in a female group than other females (*aggregation*). This effect was not significant for the whole period of lek attendance (M10, Table 3, *p*[*Dist*] = 0.08), but it became significant when we considered only the first observation at lek (M11, Table 3). Introducing the effect of the rank of the buck or of the territory did not improve the model (ΔAICc = 3.7, ΔAICc = 0.7, respectively), and neither of these variables were significant (*p* = 0.42 and *p* = 0.25, respectively).

**Figure 3:**
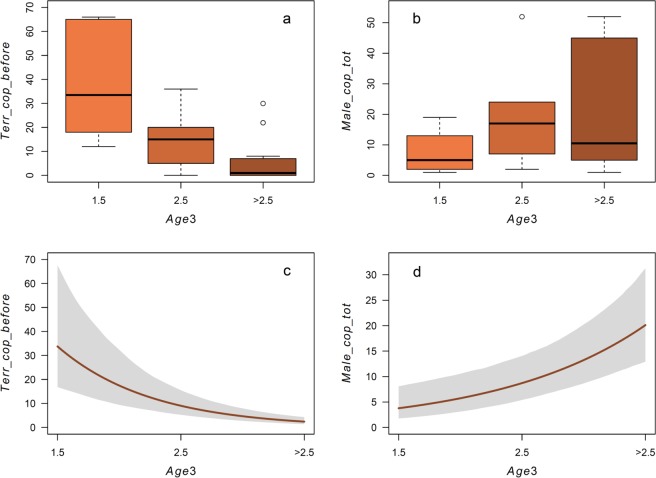
Boxplots for each age class of females mating in the lek of (**a**) the number of matings that occurred previously in the same territory from the beginning of the breeding season, and (**b**) the total copulatory success of the buck with which the female mated (bold horizontal line indicates the median, box indicates interquartile range, whiskers indicate ±1.5 × interquartile range, and outliers are shown as circles). Conditional plots (i.e., holding variable “log(*Dist*)” as constant) of Poisson GLMMs of the same relationships: (**c**) M15, (**d**) M16 (Table 3).

The previous sexual performance of the buck chosen by the tagged females (*copying*) was not related to distance or age class (M12-M13, Table 3). A similar pattern also arose when considering the mating territory instead of the buck (*territory choice*) during the same day (M14, Table 3) or from the dawn of previous day, despite in this latter analysis we noted a slight, but non-significant, negative effect of age (distance *r* = -0.26 ± 0.54, *p* = 0.65; age *r* = -0.82 ± 0.43, *p* = 0.10). Instead, the number of copulations recorded in the territory from the beginning of the mating season to the time of copulation depended on age and it was higher for the younger females (M15, Table 3; Fig. 3a,c).

#### H3. Mate quality

Contrary to expectations, considering the whole mating season, adult females were able to mate with higher ranking bucks than younger females, while there was no effect of distance (M16, Table 3; Fig. 3b,d).

## Discussion

The present analysis showed that female fallow deer adopt different tactics of mate choice in a lek and that variation in the mating tactics are associated with female experience and costs: females, who were either less experienced or incurred higher travel costs more often adopted indirect forms of mate selection when compared to adult females residing near the lek. These results address a gap in the literature: only a small number of papers in fact have dealt with female mating tactics in lekking ungulates^16,30,34^, since females are believed to be especially constrained by the resources used for rearing and protecting offsprings^14,49,50^.

The large number of visits to the lek recorded in this study for some of the females was never observed before in other fallow deer leks^9^ (where however yearling females were not investigated). Visits usually precede the copulation date, in particular for adult females. The mean time interval between successive visits is longer than the duration of the oestrus (2 days) but shorter than the oestrus cycle (22 days^51^), thus multiple visits likely represent an opportunity for male evaluation rather than mating occasions, as was previously argued^9^.

We found that younger females remained longer in the lek, performed more visits, were observed in a higher number of territories and longer in the vicinity of bucks than adults and returned to the lek also after copulating, fully confirming hypotheses H1c,d,h (i.e. more experienced females were expected, respectively, to perform less visits to the lek, to visit a lower number of male territories, and to return to the lek less frequently after copulation). Younger (1–2 year old) females were also the only ones to be recorded copulating more than once, however our limited sample (3 out of 29 tagged females) does not allow us to generalize this observation, and besides, a previous study found that fallow deer polyandry is not related to female age in a non-lekking population^37^. For younger females several visits to the lek when not in oestrus and after copulation likely imply a large energetic expenditure without an immediate gain. Overall, this apparently inefficient behaviour might be a by-product of their lack of experience and thus it can act as a learning opportunity. Further, yearling females have no fawn at heel and their movements are less constrained than the ones of adult females^50^. Multiple visits could favour mate quality assessment^9^, however younger females did not arrive earlier than older ones, albeit males were already present and available to be assessed (cf. Fig. S3) confirming hypothesis H1e (i.e. younger females were expected to arrive at the lek when mating activity has started and hence not before adult females). We might therefore deduce that yearling females are mainly interested in observing the behaviour of other females at lek and in fact they were characterised by a later copulation date, as predicted by H1f (i.e. experienced females were expected to copulate earlier). Nevertheless, we found no evidence of a higher probability of copying in younger females, contrary to hypothesis H2b (i.e. we expected that less experienced females were more likely to copy the mate choice of other females). A later copulation date of younger does could be explained by a difference in reproductive physiology between yearling and adult females^52^. This could explain why the same has been observed in a non-lekking population of fallow deer^36^. Alternatively, females of different ages might respond differently to social stimuli: females can, in fact, adjust the timing of oestrus to maximise the possibility of mating with the favourite male^35^. As a consequence, when compared to adult females, younger females could be ready to make their mate choice only after a longer assessment of males at the lek.

Despite the time spent at the lek, it appears that younger females were not able to select the highest rank bucks, contrary to hypothesis H3 (i.e. secondary tactics could increase the precision in mate assessment, therefore females that are more likely to use them should mate with highly successful males). Probably younger females were not able to identify high-ranking bucks because of their inexperience in assessing male physical traits or in recognizing visual and olfactory marking activities that are supposed to be an important signalling of male reproductive status^53^. Also in a non-lekking population, yearling females were found to mate on average with bucks of lower rank than adult ones^36^, indicating that this inability of younger females is widespread in fallow deer populations regardless the adopted mating system. Alternatively, younger females may choose to mate with lower rank males, because they are not able to pay the potential costs associated with mating with high quality males, such as aggression from other females, or the resources required to produce offspring from large males^36^. We cannot completely rule out this possibility, however the energetic expenditure associated to the large number of visits at lek and the preference for successful territories, as well as the fact that not all younger females mated with lower ranking males, suggest that our result is likely due to inexperience.

Females living far away from the lek compensate for higher costs by spending less time, visiting less often and arriving later to the lek than closer females, confirming predictions H1a (i.e. females which incurred in lower travel costs were expected to perform more visits to the lek and to arrive earlier). Despite these constraints, there is no difference between farther and near females with respect to the number of visited territories and time spent in the vicinity of bucks, thus rejecting predictions H1b (i.e. closer females were also expected to visit more male territories), as well as in copulation date. Farther females appear to use *aggregation* more than near females, though, as assumed by hypothesis H2a. Experimental studies showed that female fallow deer are attracted un-specifically by female groups^34^ and these authors argued that this behaviour is probably useful to reduce harassment by immature males and that it allows females to copy the mate choice of other does^3,14^. However, contrary to predictions H2b-c, we did not find evidence for a higher probability of *copying* or *territory choice* in farther females, therefore *aggregation* is likely to represent a cheap proxy for high ranking bucks, thus allowing the narrowing of direct assessment to only the small number of males with a harem. The final outcome is that the probability of choosing a successful male is independent of distance, suggesting that farther females are more efficient than close females, thanks perhaps to the adoption of *aggregation* behaviour. Surprisingly, younger females, when compared to older ones, were not more likely to be observed together with other females, contrary to hypothesis H2a, confirming that yearling females are not as efficient in mate selection as adult females. We cannot exclude that younger females are more likely to be harassed by subadult males than adult does, but if this is true it would be the evidence that *aggregation* was not used as a tactic to avoid harassment.

The fact that far and near females mate on average with bucks of same rank, suggests a mechanism for the origin of multiple arenas which are present in some populations^9,54^. Regarding the increased costs of a longer distance between the home range and the lek, these can be buffered by modifications in female behaviour. As we have shown in this paper, almost the whole population can congregate in a single lek, but we expect that when farther females are no longer able to compensate travel costs they are more likely to congregate in a different but nearer arena. We believe that in the case of Castelporziano, we are at the limit of a size where a single lek can be present; indeed the farthest females of our samples were in a distance class of 8.5 km and not every year were able to attain the lek. Since no other arenas have been identified in our study area (probably because the studied lek is able to attract the large majority of individuals) it is likely that females who did not reach the arena mated with males adopting strategies other than lekking (e.g. territory holding). Living far from lek may be detrimental for females (if we assume that comparing numerous males leads to a better mate choice) but this disadvantage could be counterbalanced by the benefits of philopatry^55^. In relatively larger areas, the presence of multiple arenas could be more advantageous. The critical distance threshold, though, is not expected to be the same in every study area since travel costs depend in a complex way on topography, vegetation type, physical barriers (roads, channels), presence of predators, hunting and disturbance. We also expect that variation in the condition of the study area may change, even largely, the trade-offs we have investigated.

Mate choice by females based on the selection of successful territories is widespread in lekking species (fallow deer^15^, Uganda kob^40^, blackbuck *Antilope cervicapra*^56^, black grouse^27^, great snipe *Gallinago media*^57^). This is because the location of a territory within the lek can be an honest signal of male quality, enabling less costly mate-sampling and potentially more accurate mate choice than direct female mate assessment^27^. A novel result in our study is that younger females were more likely to select a successful territory (*territory choice*) than older females confirming hypothesis H2c. Yearlings could use this tactic to increase the probability of mating with a high quality buck but this study has shown that such an approach is on average unsuccessful, probably because of the rapid turnover of bucks on the better territories. Thus, the increase in male reproductive variance in the post-*Peak* period (confirming a larger consensus of female choice later in the season, prediction H1g) can be explained by the adoption of *territory choice* by yearlings, which mate later than adult females.

Contrary to hypothesis H2b we were unable to detect a different amount of *copying* in younger or in farther females. This observation had been unexpected because previous studies^25,41,58^ have suggested that copying can be an effective tactic for cost-reducing and increasing precision. This finding concords with^34^ who found aggregation, but not copying, in female fallow deer under experimental conditions (oestrus females were offered the choice between small paddocks containing males with groups of females and males without females, or males that they had seen mating and males that they had not seen to mate). The review by Vakirtzis^59^ evidences that the presence of copying is variable in lekking species. Pruett-Jones^58^ used a theoretical game to show that it is convenient for females to copy each other’s mating decisions only when the costs of searching are not negligible. Maybe this is not the case at Castelporziano, an undisturbed area with no obstacles to animals’ movement. Gibson and Höglund^25^ argued that the benefits of imitation increase if the individuals that mate first are the ones with the greatest experience, and this is why we have originally hypothesized a more frequent *copying* in younger females. Losey *et al*.^41^ have shown that the benefit of copying increases with the number of “peeks” at lek and indeed younger females stay longer at the lek than adults. However, younger females can be deterred from copying mate choice by a rapid male turnover in territories. On the other hand, farther females cannot spend enough time in the lek to get enough peeks. A further argument which can explain the lack of differences in copying between near and farther females is males’ sperm depletion, more likely in bucks who have mated earlier^25^. Insemination failure is a problem especially for farther females, which would incur higher costs than near ones if obliged to come back to the lek later.

Our results suggest, but do not prove, that adult females residing near the lek probably perform a direct choice more than farther females. Indeed they stayed longer, visited more often and arrived earlier as predicted by H1. We can explain this pattern assuming that direct choice is especially complex and requires a careful assessment of both physical features and behaviour of bucks. In other species, direct choice is used more than indirect tactics. This is the case of the great snipe^57^ and of the non-lekking pied flycatcher *Ficedula hypoleuca*^60^. Since fallow deer exhibit a strong plasticity in the mating system^61^ which also depends on population density^62^ we can expect that direct choice may vary among populations and be more frequent in low density populations.

In general, we cannot say to what extent our results can be applied to other lekking populations of fallow deer, since relying on only one study area can introduce biases due to, e.g., geographical characteristics or management of the population, which could affect both individual spatial behaviour and demography. However, the strong behavioural difference between young and adult females found here is probably present in different populations as well, despite differences in mating system, ecology, and management.

This study demonstrated the condition-dependent variation in female mating decisions in an ungulate lek, a crucial issue to improve the understanding of mammalian mating systems^63^ and already shown in a wide range of taxa^39^. Our results can thus contribute to further clarifying the basis of the co-evolution of mating strategies in both sexes and, eventually, the evolution of leks. A main limitation of this study was that we were unable to account for the constraints determined by male behaviour on female tactics (for instance, we did not considered the effect of herding on female choice), an argument which could be the subject of further investigations.

## Supplementary information

Supplementary figures.

## Data Availability

The datasets generated during and/or analysed during the current study are available from the corresponding author upon request.
