# Supplementary material for: Female mating tactics in lekking fallow deer (Dama dama): experience explains inter-individual variability more than costs
Source: Sci Rep. 2020 Feb 27;10:3598. doi: 10.1038/s41598-020-58681-5 (PMC7046612; doi:10.1038/s41598-020-58681-5)
Supplement: Supplementary file 1 — Supplementary figures. [file 41598_2020_58681_MOESM1_ESM.pdf]

# Supplementary material

## Female mating tactics in lekking fallow deer (*Dama dama*): experience explains inter-individual variability more than costs

Imperio S., Lombardi S., De Marinis A., Ronchi F., Santini G., Focardi S.

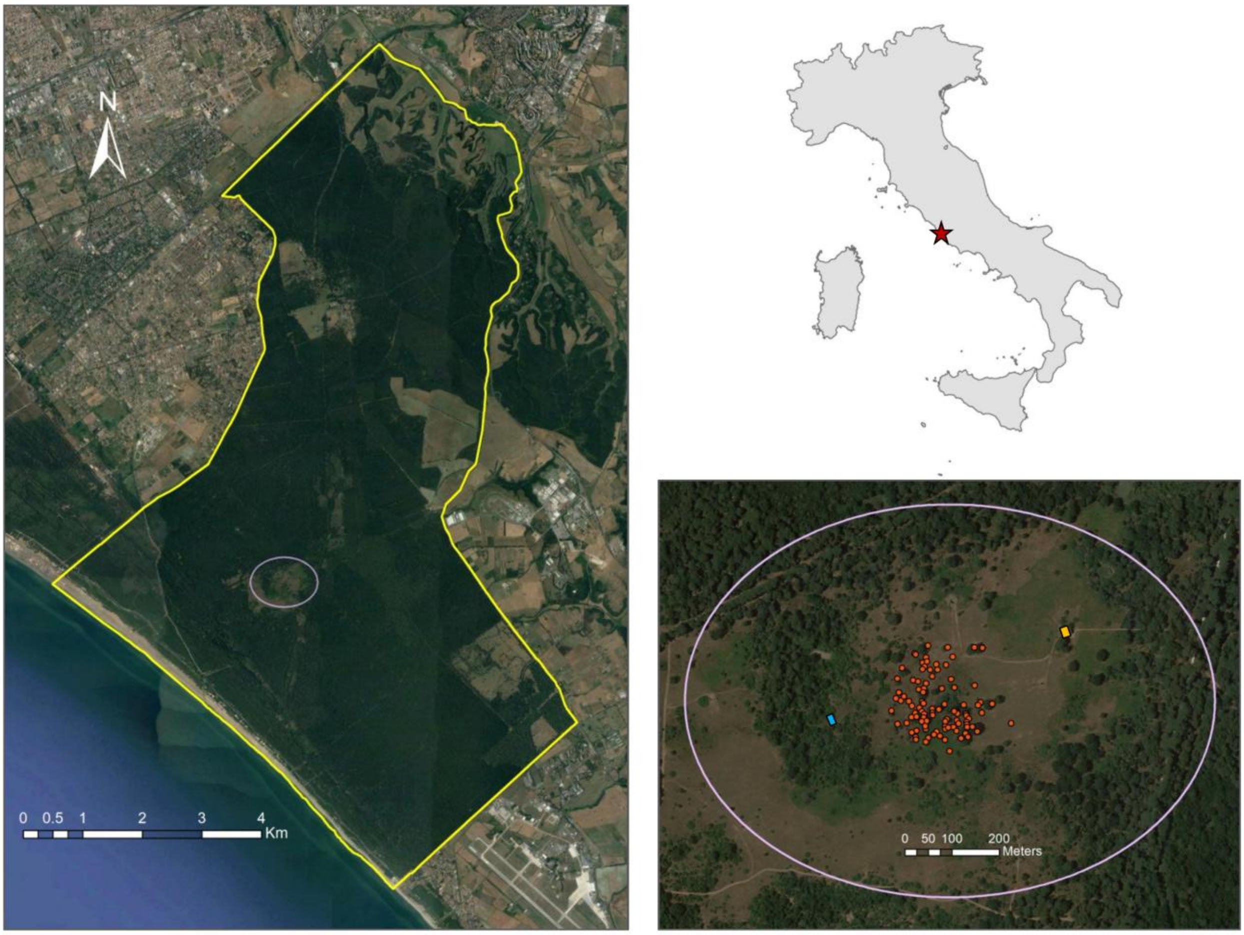


**Figure S1.** The Preserve of Castelporziano is a fenced area located near Rome (Italy). Left panel: the Preserve boundaries are outlined with a yellow line, while the lilac ellipse represents the lek area. Bottom right: a close-up of the lek; the two rectangles represent the observation points (blue = blind, orange = high seat), and red points indicate display territories of fallow deer bucks. Satellite images were downloaded from Google Earth Pro ver. 7.3.2.5776 (© 2018 Google; Data SIO, NOAA, U.S. Navy, NGA, GEBCO) and elaborated with ArcGIS ver. 10.2.2 for Desktop.

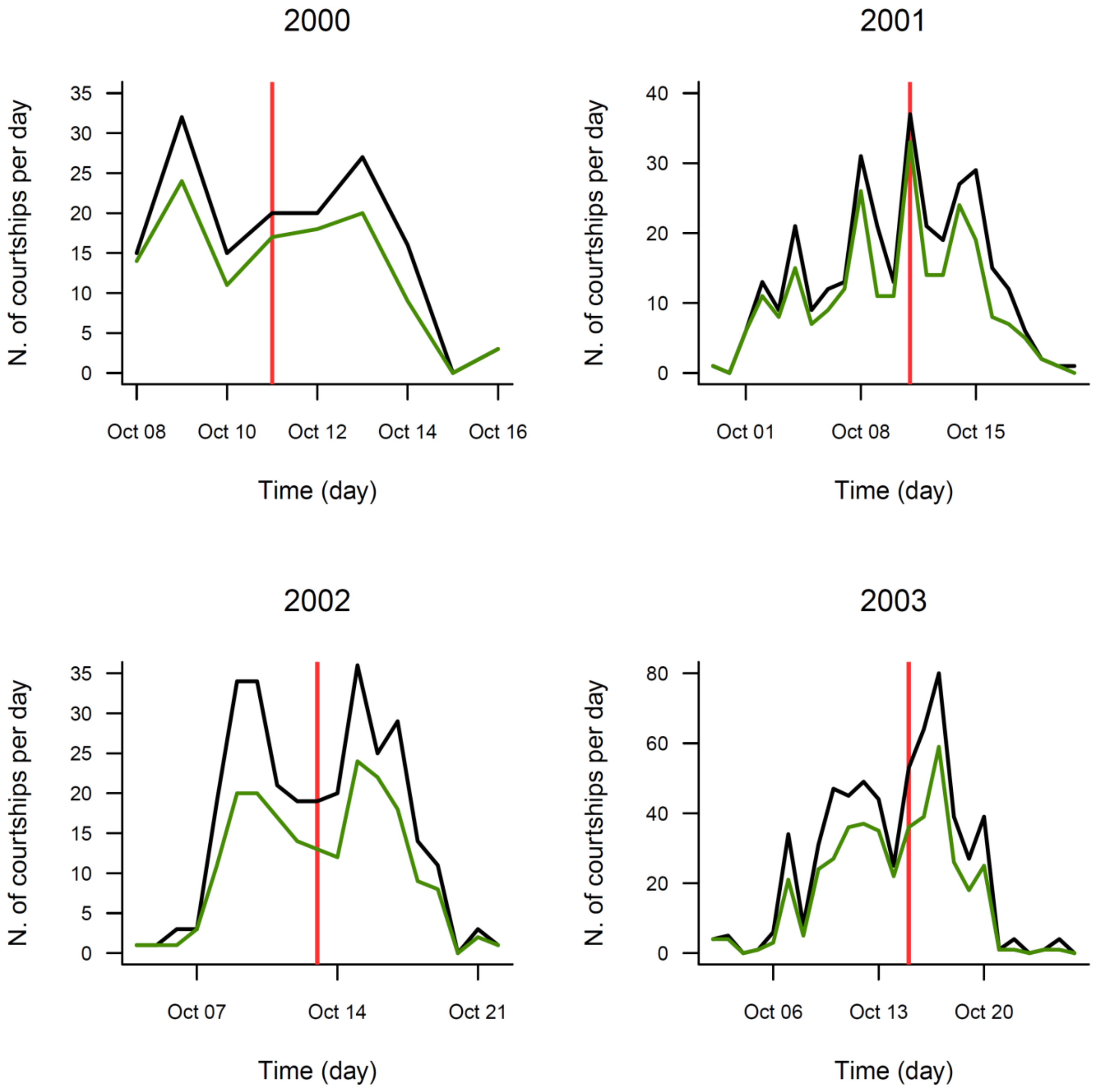


**Figure S2**. Distribution of male copulatory success at lek in the breeding seasons 2000-2003 in the Preserve of Castelporziano, Italy. Green lines represent the number of copulations (*Ejac*) per day; black lines represent the total number of courtships (*Ejac*+*IntC*) per day; the *Peak* date is indicated with a red vertical line.

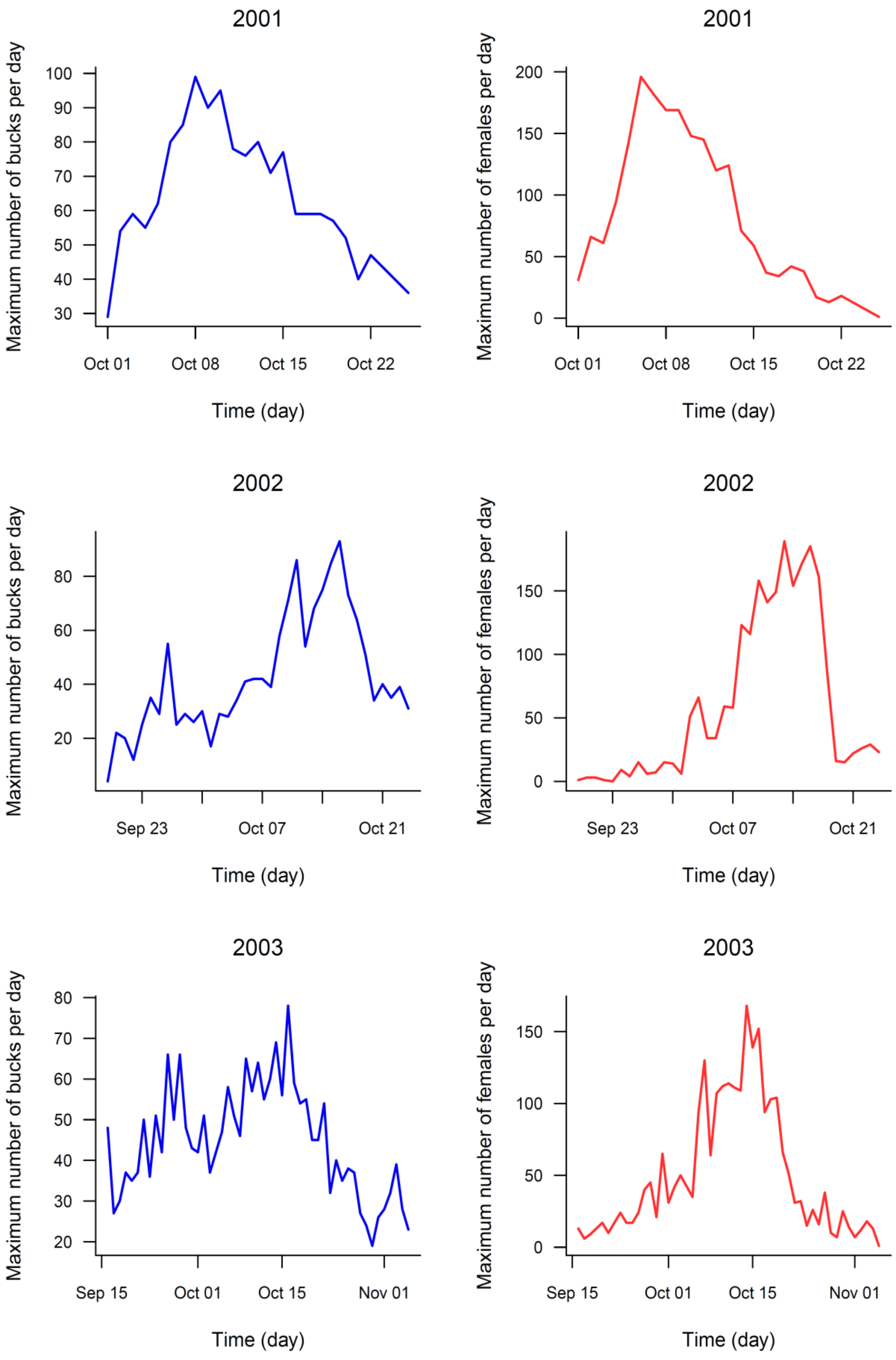


**Figure S3**. Daily number of bucks and does present in the lek during the breeding seasons 2001-2003 in the Preserve of Castelporziano, Italy.
